# Reprogramming tumor-associated macrophages in gastric cancer: a pathway to enhanced immunotherapy

**DOI:** 10.3389/fimmu.2025.1558091

**Published:** 2025-03-03

**Authors:** Yibo He, Qianran Hong, Shiliang Chen, Jiayi Zhou, Shengliang Qiu

**Affiliations:** The First Affiliated Hospital of Zhejiang Chinese Medical University (Zhejiang Provincial Hospital of Chinese Medicine), Hangzhou, Zhejiang, China

**Keywords:** gastric cancer, tumor microenvironment, tumor-associated macrophages, polarization of macrophages, immunotherapy

## Abstract

Gastric cancer (GC) remains a significant global health concern due to its poor prognosis and limited therapeutic options, particularly in advanced stages. Tumor microenvironment (TME), particularly tumor-associated macrophages (TAMs), plays a key role in tumor progression, immune evasion, and therapy resistance. TAMs exhibit plasticity, shifting between pro-inflammatory M1 and immunosuppressive M2 phenotypes, with the latter predominating in GC and contributing to poor outcomes. Recent therapeutic advancements focus on targeting TAMs, including inhibiting M2 polarization, reprogramming TAMs to M1 phenotypes, and combining TAM-targeted approaches with immune checkpoint inhibitors. Innovations in nanotechnology, metabolic reprogramming, and targeting key pathways such as interleukin-6 and C-C motif ligand 2/C-C motif chemokine receptor 2 further enhance these strategies. However, challenges remain, including the spatial and functional heterogeneity of TAMs within the TME and the need for selective targeting to avoid disrupting immune homeostasis. Ongoing research on TAM origins, functions, and interactions within the TME is crucial for developing precise and effective therapies. These advances hold promise not only for improving outcomes in GC but also for addressing other cancers with similarly complex microenvironments.

## 1 Introduction

Gastric cancer (GC) is a major global health concern, ranking as the third leading cause of cancer-related mortality and the fifth most common cancer worldwide. Its poor prognosis is attributed to late-stage diagnoses and limited therapeutic options, as most patients present with advanced disease, where traditional treatments like surgery and chemotherapy offer modest survival benefits (1, 2). Although immunotherapy has revolutionized cancer treatment, its efficacy in GC remains limited due to the complexity of the tumor microenvironment (TME), which fosters immune evasion and resistance (3). Immune checkpoint inhibitors (ICIs), such as nivolumab and pembrolizumab, have shown significant survival benefits in specific subgroups, particularly in patients with microsatellite instability (MSI) or Epstein-Barr virus-positive tumors, but most GC patients fail to respond effectively (4). Challenges like identifying reliable predictive biomarkers, such as programmed cell death-ligand 1 (PD-L1) expression and tumor mutational burden, further limit the precision of immunotherapy application (5–7). Current research focuses on overcoming these hurdles by exploring combination therapies, such as ICIs paired with chemotherapy or targeted agents, which have shown promising results, especially in human epidermal growth factor receptor 2 (HER2)-positive GC patients (2, 8). Additionally, novel strategies like chimeric antigen receptor T-cell therapy and cancer vaccines are under development, offering hope for improved outcomes. However, addressing the complexities of the TME and refining patient stratification through biomarkers remain critical for advancing GC immunotherapy.

TME is a complex network of tumor cells, immune cells, stromal cells, and the extracellular matrix that significantly influences tumor progression and its dynamics are closely related to cancer (9, 10). Among its components, tumor-associated macrophages (TAMs) are key regulators, playing dual roles. M1 macrophages exhibit anti-tumor activity, while M2 macrophages promote tumor growth, immune suppression, and metastasis (11, 12). High M2 TAM infiltration is linked to poor prognosis and enhanced immune evasion, often mediated through upregulated immune checkpoint proteins like PD-L1, which reduce T-cell activity (13, 14). Furthermore, TAMs correlate strongly with GC stage, metastasis, and resistance to therapies, making them critical players in tumor progression (15). TAMs also interact with stromal components like cancer-associated fibroblasts (CAFs) and mesenchymal stromal cells, which polarize macrophages to the M2 phenotype and enhance tumorigenic processes such as epithelial-mesenchymal transition (EMT) and metastasis (16, 17). These interactions highlight the dynamic nature of the TME and its role in promoting tumor resistance. Recent studies suggest that targeting TAMs-associated pathways, such as the IL-6/interleukin-8 (IL-8) axis or lactate-monocarboxylate transporter-hypoxia-inducible factor 1 alpha (HIF1α) signaling pathway, could reprogram macrophages and disrupt their pro-tumor activities (16, 18). These findings emphasize the potential of modulating the TME, particularly TAMs, to enhance the efficacy of GC immunotherapy.

TAMs play a multifaceted role in GC, influencing immune evasion, metastasis, and treatment resistance. TAMs exhibit plasticity, adopting either pro-inflammatory (M1) or tumor-promoting (M2) phenotypes, with the latter dominating in GC. M2 TAMs contribute to immune suppression by upregulating PD-L1, limiting T-cell responses, and promoting angiogenesis and EMT via cytokines like IL-6 and IL-8 (15, 16). This polarization correlates with advanced tumor stage, poor prognosis, and chemotherapy resistance, underscoring the clinical significance of TAMs (19). Emerging studies highlight the potential to target TAMs therapeutically. Strategies include inhibiting M2 polarization, blocking TAM-derived signals such as vascular endothelial growth factor-A, and reprogramming TAMs into the M1 phenotype. For example, SPI1-positive CD68-positive Macrophages (SPI1+CD68+ macrophages) have been identified as markers for metastasis and potential targets for combined immunotherapy and anti-angiogenic treatment (20). Additionally, disrupting exosomal communication, such as miR-487a transfer from TAMs to tumor cells, has shown promise in reducing metastasis and tumor growth (21). These advances demonstrate the critical role of TAMs in GC progression and their potential as therapeutic targets.

## 2 Origin and classification of TAMs

### 2.1 Dual origin of TAMs: monocyte recruitment and embryonic-derived macrophages

TAMs in TME originate from two primary sources: monocytes recruited from the peripheral blood and tissue-resident macrophages derived from embryonic progenitors. Circulating monocytes, under the influence of chemokines like C-C motif ligand 2 (CCL2) and C-C motif ligand 5 (CCL5), are actively recruited to the tumor site, where they differentiate into macrophages and adopt pro- or anti-tumor phenotypes depending on microenvironmental cues (22). These monocyte-derived macrophages are highly plastic and play a significant role in immune suppression, tumor invasion, and angiogenesis (23). On the other hand, tissue-resident macrophages such as Kupffer cells in the liver or microglia in the brain originate from embryonic yolk sac progenitors and persist throughout life, maintaining their population through local self-renewal (24). These embryonic macrophages exhibit distinct transcriptional profiles and contribute to tissue remodeling and immunosuppressive niches that support tumor progression (25).

Recent studies have highlighted the interplay between these two macrophage populations in the TME. While monocyte-derived macrophages dominate in inflamed or late-stage tumors, embryonic-derived macrophages are often involved in the early stages of tumorigenesis, facilitating immune evasion and stromal remodeling (26). For instance, embryonic macrophages, by virtue of their tissue-specific adaptations, play a critical role in maintaining homeostasis and promoting metastasis in certain cancers, including GC (27). This dual origin and functional heterogeneity of TAMs complicate therapeutic targeting but also offer opportunities to develop precise, lineage-specific interventions. Understanding TAM ontogeny is vital for designing effective therapeutic strategies. Targeting monocyte recruitment pathways, such as C-C motif ligand 2/C-C motif chemokine receptor 2 (CCL2/CCR2), has shown promise in reducing tumor infiltration by monocyte-derived macrophages (22). Similarly, interventions aimed at reprogramming embryonic macrophages to adopt anti-tumor phenotypes are under investigation. Such approaches could enhance the effectiveness of immunotherapy and reduce tumor-promoting functions of TAMs.

### 2.2 Functional states of TAMs in gastric cancer (M1 vs. M2 phenotype)

**Figure 1:**
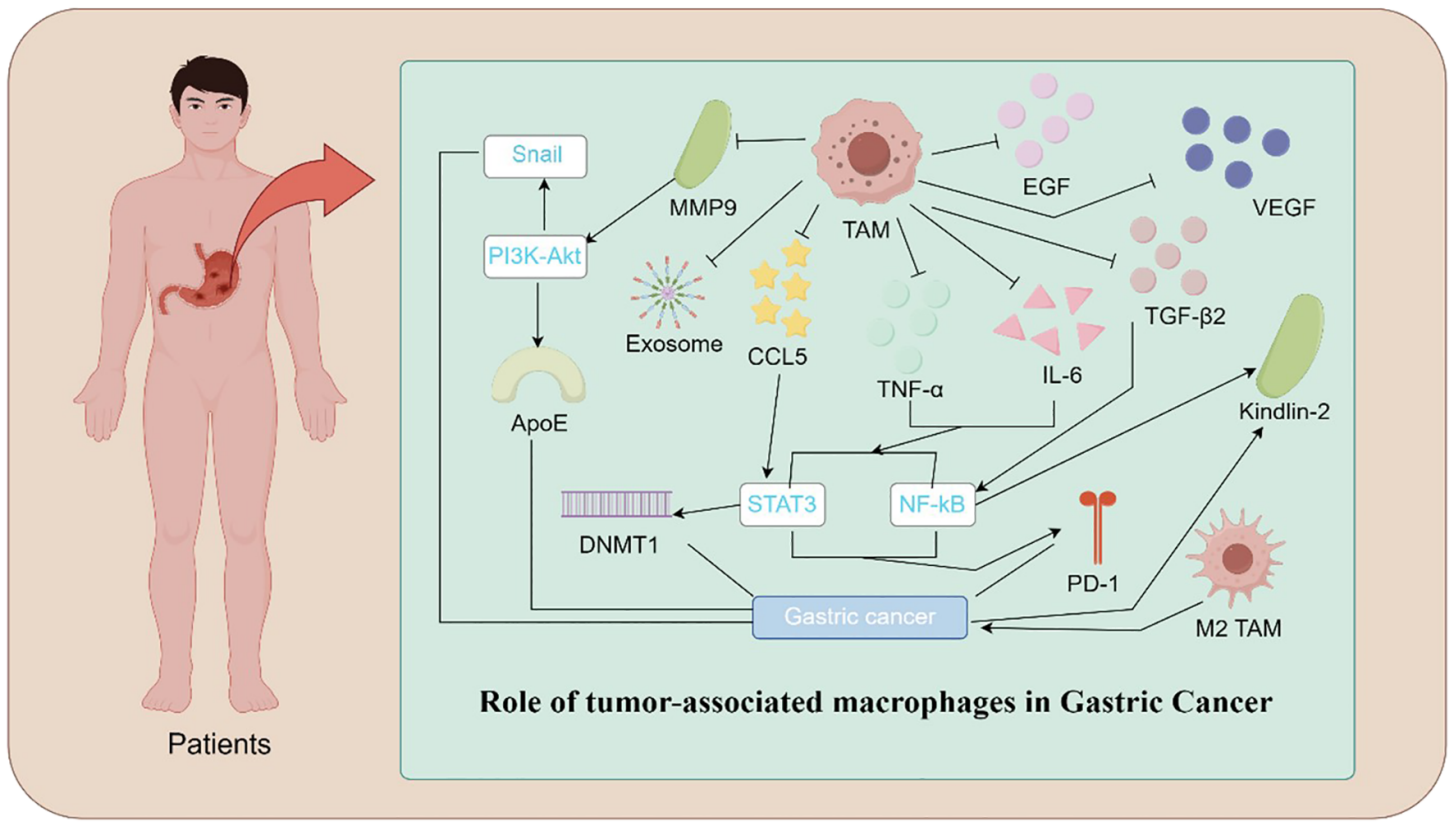
Mechanistic Illustration of TAMs in Gastric Cancer. Illustration depicting the role of TAMs in gastric cancer progression. TAMs contribute to tumor growth and metastasis through various mechanisms. These include the release of pro-tumorigenic factors such as IL-6, TNF-α, VEGF, TGFβ2, and EGF, promoting angiogenesis and immune evasion. TAM-secreted exosomes and CCL5 activate signaling pathways, including PI3K-Akt, STAT3, and NF-κB, which enhance cancer cell proliferation and invasion. Additionally, epigenetic modifications mediated by DNMT1 and molecules such as ApoE and Kindlin-2 facilitate tumor progression. Polarization of TAMs toward the M2 phenotype and the upregulation of PD-1 further suppress anti-tumor immune responses, highlighting the central role of TAMs in the gastric cancer microenvironment.

During the early stages of GC development, the inflammatory response in the lesion area is predominantly guided by T Helper 1 (T_H_1) cells and T Helper 2 (T_H_2) cells, which respectively drive macrophages towards M1 and M2 polarization (28). M1 macrophages exert anti-tumor effects by activating T cells through the release of cytokines such as interleukin-12 (IL-12) and TNF-α. In contrast, M2 macrophages promote tumor progression by secreting anti-inflammatory factors like interleukin-10 (IL-10), which not only promote angiogenesis and suppress immune responses but also facilitate EMT (29, 30). TME of GC, characterized by hypoxia, metabolic alterations, and immunosuppressive cytokines, predominantly exhibits an M2 phenotype in TAMs, a condition that fosters immune escape and tumor growth, significantly correlating with poor patient prognosis (12, 31) (**Figure 1**).

The polarization of macrophages in GC is influenced by a complex interplay of signaling pathways, transcription factors, and epigenetic regulation (32). The janus kinase/signal transducers and activators of transcription signaling pathway plays a pivotal role in the polarization of M2 macrophages, mediated by cytokines such as interleukin-4 (IL-4), IL-6, and interleukin-13 (IL-13), which modulate immune responses and facilitate tumor escape. Concurrently, the nuclear factor kappa-B (NF-κB) signaling pathway is crucial for the pro-inflammatory activation of M1 macrophages, closely associated with inflammation and cancer progression in GC. The transcription factor HIF1α supports tumor growth by promoting angiogenesis in the hypoxic TME. Additionally, DNA methylation and histone modifications regulate macrophage gene expression, while microRNAs such as miR-146a and miR-155 fine-tune the immune microenvironment of GC.

Further research reveals that the activation mechanism of M1 in GC involves pro-inflammatory cytokines secreted by GC cells, such as interferon gamma (IFN-γ), stromal factors like GM-CSF, and pathogenic molecules such as lipopolysaccharides present in the TME (28). However, M1 macrophages are often forced to shift toward the M2 phenotype due to the influence of factors such as IL-4, IL-13, transforming growth factor-β1 (TGFβ1), arginase 1, nitric oxide, and long non-coding RNAs (12). Additionally, the hypoxic conditions in the TME drive the M1-to-M2 transition through HIF1α signaling and metabolic reprogramming, which includes glycolysis and lactate production (18). Furthermore, tumor-derived hyaluronan fragments contribute to the development of M2 macrophages, and interactions between tumor cells and stromal components, such as CAFs, help maintain the M2 (29). These factors collectively lead to an increased proportion of M2 in the TME, thus shaping a TME that promotes GC progression. The M1/M2 ratio has emerged as a potential prognostic marker for assessing the progression and treatment resistance of GC, offering new perspectives and potential therapeutic targets for the treatment and pathological study of GC (33).

Therapeutically, targeting TAM polarization has become a promising strategy to improve anti-tumor immunity in GC. Approaches include inhibiting the recruitment of M2 TAMs, blocking signaling pathways such as lnterleukin-6/signal transducer and activator of transcription 3 (IL-6/STAT3), and reprogramming M2 macrophages into the M1 phenotype using agents like STAT6 inhibitors and metabolic modulators (30, 34). These interventions aim to alter the balance within the TME, restore immune surveillance, and enhance the efficacy of immunotherapies such as checkpoint inhibitors. However, the precise and selective targeting of M2 to activate immune responses in cancer lesion areas remains a challenge, necessitating further research.

### 2.3 Dynamic distribution and heterogeneity of TAMs in the gastric

Cancer Microenvironment TAMs in GC are highly heterogeneous, with their distribution varying significantly depending on tumor regions, such as the tumor core (TC) versus invasive margins (IM). This spatial heterogeneity is closely linked to their distinct phenotypic and functional characteristics. M2-polarized TAMs, known for their pro-tumorigenic role, tend to accumulate at the IM, where they promote immune evasion and metastasis, while a mix of M1 and M2 TAMs is often observed within the TC (35, 36). These regional differences reflect the diverse microenvironmental cues driving TAM polarization, including hypoxia, tumor cell interactions, and cytokine gradients (37). Notably, higher densities of M2 TAMs at the IM are associated with poor prognosis and reduced overall survival in GC patients, highlighting their clinical relevance (19). The heterogeneity of TAMs also impacts their interactions with other immune cells. For instance, TAMs at the IM often suppress CD8+ T-cell activity by secreting immunosuppressive factors such as IL-10 and TGFβ, facilitating immune escape (37). Conversely, TAMs within the TC may exhibit a more mixed phenotype, with M1 TAMs supporting anti-tumor immunity under specific conditions. Advanced techniques, such as multiplex immunohistochemistry and single-cell RNA sequencing, have been instrumental in uncovering these spatial patterns, revealing TAMs’ dynamic roles within different tumor compartments (20, 36). Therapeutically, targeting the spatial heterogeneity of TAMs offers new opportunities for improving GC outcomes. Strategies that selectively deplete or reprogram M2 TAMs at the IM, while preserving M1 TAM functions within the TC, could enhance anti-tumor immunity and synergize with existing therapies such as ICIs (20, 30). Understanding the spatial and functional diversity of TAMs is thus critical for developing effective, targeted immunotherapies in GC.

## 3 Interactions between TAMs and the immune microenvironment in gastric cancer

### 3.1 How TAMs remodel the gastric cancer immune microenvironment

TAMs play a critical role in shaping the immune microenvironment in GC, where they regulate immune responses, immune cell activity, and tumor metabolism. M1 macrophages promote the infiltration of CD8+ T cells and natural killer (NK) cells by secreting cytokines and chemokines (28), thereby enhancing immune responses. However, in the GC immune microenvironment, M2 macrophages dominate. These cells primarily suppress CD8+ T cell function by secreting immune-suppressive factors such as IL-10 and TGFβ, facilitating tumor immune evasion (12). Additionally, M2 macrophages further exacerbate the immune-suppressive environment by enhancing the infiltration of regulatory T cells (18, 38). Tumor-derived exosomes also regulate TAM polarization, promoting their conversion to the M2 phenotype. This process enhances angiogenesis and tissue remodeling while suppressing the activity of cytotoxic immune cells, thus further driving immune evasion (17). Dickkopf-1 (DKK1) has been identified as another regulator of TAM activity, promoting an immunosuppressive phenotype that hinders CD8+ T cells and NK cells while facilitating immune evasion (14).Hypoxic conditions and Met-enkephalin drugs also influence macrophage polarization, further promoting immune suppression. Therefore, TAMs are not only key immune modulators in the tumor microenvironment but also potential therapeutic targets in immunotherapy.

The interactions of various signaling pathways play an essential role in the shaping of the immune microenvironment by TAMs. TAMs enhance tumor immune evasion by interacting with immune checkpoint pathways, such as programmed cell death protein 1/programmed cell death ligand 1 (PD-1/PD-L1) axis. High PD-L1 expression in TAMs is closely associated with poor prognosis, with PD-L1+ TAMs suppressing the immune function of CD8+ T cells and weakening anti-tumor immune responses (39). The janus kinase/signal transducer and activator of transcription (JAK/STAT) signaling pathway is particularly important in the GC immune microenvironment. Studies have shown that the abnormal activation of the JAK/STAT pathway in a GC peritoneal dissemination model is closely associated with resistance to ICIs, with M2 macrophage infiltration aggravating the immune-suppressive environment (40). Moreover, the JAK/STAT pathway regulates the thioredoxin interacting protein-reactive oxygen species axis, influencing TAM polarization and playing a pivotal role in shaping the GC immune microenvironment (41). C-X-C motif chemokine ligand-5 recruits monocytes, further promoting the formation of more M2 macrophages and activating the phosphoinositide 3-kinase/protein kinase B/mechanistic target of rapamycin (PI3K/AKT/mTOR) pathway, which contributes to the development of GC chemotherapy resistance (42). The wingless-related integration site (Wnt) signaling pathway promotes macrophage polarization to the M2 phenotype via both the Wnt/β-catenin and non-canonical Wnt/Ca²⁺ pathways, accelerating tumor growth and metastasis (43). The NF-κB pathway enhances M2 TAM polarization, reinforcing their immune-suppressive function and tumor cell survival, thereby accelerating the progression of precancerous lesions (44). The TGFβ pathway regulates TAM polarization in the GC immune microenvironment and affects the stem cell properties of GC cells (45). Furthermore, the HIF-α signaling pathway regulates macrophage polarization to the M2 phenotype through the hypoxia-glycolysis axis, inhibiting T cell function and promoting immune evasion and resistance (46). The IL-4/IL-13 pathway induces M2 polarization in macrophages, enhancing immune suppression and angiogenesis (47). The PI3K/AKT/mTOR pathway mediates exosome-induced remodeling of the GC cell cytoskeleton (47). The CSF-1/CSF-1R pathway facilitates the conversion of M1 macrophages to M2 macrophages, while the TGFβ1 pathway suppresses NK cells and induces EMT (47).

### 3.3 How TAMs intervene gastric cancer progression

TAMs significantly influence the development of GC by accelerating tumor progression and exacerbation across multiple dimensions (32). Initially, TAMs support tumor growth by promoting angiogenesis and neovascularization, supplying essential nutrients and oxygen to the tumor. This process involves the secretion of inflammatory cytokines (such as IL-4 and IL-10) and vascular endothelial growth factor (VEGF), which activate relevant signaling pathways, such as NF-kB, thereby promoting the formation of blood vessels and the survival of tumor cells. Additionally, TAMs cooperate with CAFs in the remodeling of the extracellular matrix and tissue, accelerating tumor cell invasion and migration by secreting proteases such as matrix metalloproteinases and cathepsins. In terms of tissue invasion and distant metastasis, TAMs enhance the invasiveness and migratory capacity of tumor cells by activating the EMT, particularly through the activation of toll-like receptor-4 and the subsequent secretion of cytokines such as IL-6 and TNF-α. Moreover, M1-like TAMs exhibit the potential to suppress tumors by activating immune responses and promoting the proliferation and functionality of T cells. Furthermore, the role of TAMs in tumor self-renewal is crucial, particularly their interactions with cancer stem cells, notably through promoting the epidermal growth factor (EGF) signaling pathway, which supports the survival and self-renewal of tumor cells, thereby enhancing tumor persistence and treatment resistance.

In summary, TAMs play a pivotal role at various stages of GC development through these cellular and molecular mechanisms, not only facilitating tumor growth and angiogenesis but also significantly contributing to the remodeling of the extracellular matrix, tissue invasion, and distant metastasis.

### 3.4 Regulation of immune checkpoint molecules by TAMs

TAMs significantly influence the regulation of immune checkpoint molecules in TME of GC. These macrophages express high levels of PD-L1, which suppresses anti-tumor T-cell activity and correlates with immune evasion (3). Studies have shown that TAMs interact with the PD-1/PD-L1 axis to directly impair CD8+ T-cell function, enabling tumors to escape immune surveillance. For instance, blocking the PD-1/PD-L1 pathway alone often results in suboptimal therapeutic responses, emphasizing the role of TAMs in checkpoint resistance (48). Additionally, TAMs mediate immune resistance through other checkpoint molecules such as V-domain Ig suppressor of T cell activation and T-cell immunoglobulin and mucin-domain containing-3, further suppressing T-cell activation and promoting a highly immunosuppressive environment (49).

TAMs also regulate the efficacy of immune checkpoint blockade therapies. Recent findings suggest that TAM polarization towards the M2 phenotype contributes to an upregulation of PD-L1 expression, which directly correlates with the resistance to checkpoint inhibitors. Targeting pathways such as the PI3K/AKT axis within TAMs has been shown to decrease their immunosuppressive phenotype, enhancing CD8+ T-cell function and boosting anti-tumor immunity (14). Moreover, studies demonstrate that therapies combining checkpoint inhibitors with TAM reprogramming agents, such as C5a receptor 1 blockade, can synergistically reinvigorate T-cell cytotoxicity and improve clinical outcomes (11, 48).

## 4 Immunotherapeutic strategies targeting TAMs

### 4.1 Overview of TAM-targeting strategies

TAMs are critical regulators of the TME, making them valuable targets for cancer immunotherapy. One common strategy is TAM depletion, primarily achieved by blocking the colony-stimulating factor 1/colony-stimulating factor 1 receptor (CSF-1/CSF-1R) signaling pathway. This pathway is essential for macrophage survival and recruitment, and inhibitors like BLZ-945 have shown efficacy in reducing TAM populations and enhancing anti-tumor immunity in preclinical models (50). Nanoparticle delivery systems targeting TAM depletion have also emerged, offering precision drug delivery while minimizing systemic toxicity (51). Furthermore, combining TAM depletion with ICIs has demonstrated synergistic effects, as seen in clinical trials with CSF-1R inhibitors in combination with anti-PD-1 therapies (52). Beyond depletion, an alternative strategy focuses on reprogramming TAMs from an immunosuppressive M2 to a tumoricidal M1. Various agents, including PI3K inhibitors, toll-like receptor agonists, and histone deacetylase inhibitors, have been studied for their ability to reprogram TAMs, thereby promoting T-cell activation and tumor cytotoxicity (53). Nanotechnology has also further advanced this approach, with M2 macrophage-targeting nanoparticles successfully polarizing TAMs toward the M1 phenotype and improving tumor immune responses (54, 55). Additionally, drugs such as zoledronic acid and resiquimod, delivered via nanoparticles, have demonstrated efficacy in reversing TAM-mediated immunosuppression (56).

Inhibiting key TAM-related pathways is another promising avenue. The CCL2/CCR2 axis, which regulates monocyte recruitment and differentiation into TAMs, has been effectively targeted to disrupt TAM function and enhance tumor sensitivity to immunotherapy (16, 57). Blocking hypoxia-related pathways within TAMs has also proven effective, as hypoxia-inducible factors promote TAM recruitment and M2 polarization. Strategies targeting hypoxia pathways combined with immunotherapies have shown synergistic effects, improving outcomes in both primary and metastatic cancers (58, 59). Moreover, targeting TAM-expressed immune checkpoints like PD-L1 has emerged as a complementary strategy, further enhancing immune checkpoint blockade therapies (54).

### 4.2 Combination therapies targeting TAMs

Targeting TAMs in combination with ICIs has shown promise in enhancing anti-tumor immunity. TAMs contribute to immune evasion by promoting a suppressive TME that inhibits T-cell function, thereby limiting the efficacy of ICIs. Studies suggest that combining TAM depletion or reprogramming with ICIs, such as anti-PD-1 or anti-PD-L1 therapies, can significantly improve outcomes. For example, TAM-targeting therapies like CSF-1R inhibitors have been shown to reduce TAM-mediated suppression, thereby sensitizing tumors to ICIs (60). Additionally, the use of TAM-targeted tyrosine kinase inhibitors in conjunction with ICIs has demonstrated synergistic effects in preclinical studies, offering a new avenue for combination therapy (61).

TAMs also play a role in modulating the effectiveness of chemotherapy and radiotherapy by creating a pro-tumorigenic environment that fosters resistance to these treatments. Strategies targeting TAMs have been shown to enhance the efficacy of conventional therapies. For instance, combining TAM depletion or reprogramming with chemotherapeutic agents such as paclitaxel can promote macrophage-mediated phagocytosis and tumor clearance (62). Similarly, radiotherapy combined with TAM modulation can alter the immunosuppressive TME and improve the recruitment of effector T cells, thereby potentiating anti-tumor responses (63).

The metabolic reprogramming of TAMs is emerging as a novel strategy to modulate their functional state. TAMs rely on distinct metabolic pathways, such as glycolysis and fatty acid oxidation, to sustain their pro-tumorigenic phenotype (64, 65). Targeting these metabolic pathways can shift TAMs toward an anti-tumorigenic phenotype, enhancing the efficacy of immunotherapies. For instance, a study demonstrated that lactate depletion combined with CRISPR-based SIRPα gene editing effectively reprogrammed TAMs and reversed the immunosuppressive TME (66). Furthermore, metabolic regulators, such as Adenosine Monophosphate-Activated Protein Kinase and Monocarboxylate Transporter 4 inhibitor, when used in conjunction with immune checkpoint blockade, have demonstrated potential in enhancing tumor immunity and improving treatment outcomes (67, 68).

### 4.3 Progress of immunotherapy in GC

Currently, radiotherapy, chemotherapy, targeted therapy, and surgery remain the main treatment modalities for GC. However, with the rapid development of immune-related research, immunotherapy is gradually reshaping the treatment pattern of GC (69, 70).

#### 4.3.1 Molecular mechanisms, immune resistance, and biomarker-driven strategies in GC immunotherapy

Recent studies have shown that targeting M2 may enhance the efficacy of anti-PD-1/PD-L1 therapies, and the expression of PD-L2 may serve as a potential biomarker to assess the optimal timing for immunotherapy (47). Moreover, targeting SPI1+CD68+ TAMs may help optimize the therapeutic effects of ICIs, such as PD-1/PD-L1 and cytotoxic T-lymphocyte antigen-4 inhibitors (20). Manipulating the stimulator of interferon genes (STING) pathway to regulate the IL-6 receptor-janus kinase-signal transducer and activator of transcription signaling pathway (JAK/STAT) in TAMs and its downstream target interleukin-24, thereby inducing a pro-inflammatory phenotype, may represent a promising strategy for GC immunotherapy (71). The PI3K-γ signaling pathway influences the immune-suppressive function of GC TAMs by regulating lipid metabolism. Inhibition of TGFβ1 can restore NK cell function, offering a potential strategy for GC treatment (47). GC cell-derived serpin family E member 1 (SERPINE1), by activating the JAK2/STAT3 signaling pathway, regulates the transfer of exosome-derived let-7g-5p, promoting M2 polarization (72). Inhibiting the function of SERPINE1 may emerge as a new target to enhance immunotherapy. The role of HIF1α in immune evasion and drug resistance in GC has attracted considerable attention (46). Factors such as signal regulatory protein alpha, tyrosine kinase with immunoglobulin-like and EGF-like domains 1, and nuclear Fe-Sulfur cluster 1 may serve as potential biomarkers for diagnosing, prognosticating, and evaluating the efficacy of immunotherapy in GC (73–75).

However, GC remains a highly lethal gastrointestinal malignancy, and its treatment faces several challenges, including immune resistance and the complex interactions within TME (76). Despite some progress with immunotherapies, such as PD-1 inhibitors, many patients exhibit resistance, which is partly attributed to the intricate interactions within the TME (77). Modulating the TME, particularly through targeted therapies that focus on specific molecular markers like ERBB2, may enhance the effects of immunotherapy and reduce resistance (78). Additionally, combining multiple ICIs has emerged as a promising strategy to overcome immune evasion and resistance in GC (79). For instance, the regulation of LOX gene expression by lncRNA and miR-29c has shown potential in modulating the tumor immune environment and chemotherapy sensitivity, offering a new therapeutic target for GC (80). Furthermore, exploring the role of the gut microbiome may provide novel insights into overcoming the limitations of current immunotherapy strategies (81). These studies highlight that a multifaceted approach to modifying the immune microenvironment may hold promise for improving the efficacy of immunotherapies and reducing resistance in GC (79, 81).

#### 4.3.2 Harnessing traditional medicine and natural products for immune microenvironment reprogramming

Traditional Chinese medicine (TCM) formulations and natural products have demonstrated significant immune-regulatory effects in the modulation of TAMs, particularly in the context of GC immunotherapy. Various TCM formulations regulate TAMs and the immune microenvironment through multiple pathways, thereby enhancing antitumor immune responses (82). For example, compound Kushen injection, when combined with FOLFOX chemotherapy, has been shown to downregulate the expression of pro-inflammatory cytokines IL-6 and TGFβ1, alleviating TAM-mediated immune suppression, while simultaneously promoting the activation of CD3^+^ and CD4^+^ T lymphocytes, and reshaping the GC immune microenvironment towards a pro-inflammatory phenotype. Buzhong Guben Yiwei Decoction enhances the proportion of CD3^+^ and CD4^+^ T cells, boosts the function of effector T cells, inhibits the secretion of immune-suppressive factors IL-10 and TGFβ1, and reduces the infiltration of Treg cells, further reversing the immune suppression dominated by TAMs, thereby providing robust microenvironmental support for GC immunotherapy. Furthermore, Astragalus polysaccharide, when used in combination with targeted therapies such as apatinib, inhibits the protein kinase b/extracellular signal-regulated kinase signaling pathway, reduces the expression of matrix metalloproteinase-9, suppresses the invasion and metastasis of GC cells, and promotes the conversion of TAMs from the M2 to M1, thereby activating the anti-tumor immune response. Melittin, at appropriate concentrations, facilitates the polarization of TAMs from the M2 to the M1 phenotype, further enhancing the immune response (47). Betulinic acid not only inhibits the expression of stemness-related proteins mediated by glucose-regulated protein 78 and macrophage polarization into TAMs through GRP78-TGFβ signaling, but also suppresses the cancer stemness induced by TAMs (45). Sophoridine, by inhibiting the immune suppressive function of M2 macrophages, enhances the cytotoxic activity of CD8+ T cells and suppresses the infiltration of macrophages into the GC microenvironment, showing promise as a potential adjuvant in immunotherapy (83).

#### 4.3.3 Nanotechnology-driven delivery systems and multimodal immunotherapeutic platforms

Nanotechnology demonstrates significant potential in enhancing immunotherapy for GC by improving antitumor immune responses and optimizing therapeutic efficacy (84). First, nanocarriers targeting TAMs, such as polymer nanoparticles loaded with TGFβ1 inhibitors, effectively reprogram the TME by restoring NK cell function (85). Ligand-modified nanoparticles further facilitate the polarization of TAMs from the pro-tumorigenic M2 to M1. For instance, Rm@PP-GA nanoparticles, utilizing an erythrocyte-mimicking delivery mechanism, precisely deliver photo-STING agonists, achieving TAM polarization to the antitumor M1 phenotype, activating the immune system, and ultimately eradicating tumors. Second, nanocarrier systems improve the delivery of immunomodulators (e.g., IL-2, IFN-γ) and chemotherapeutic agents (e.g., cisplatin, paclitaxel), enhancing drug specificity and efficacy (86). Examples include liposomes and metal nanoparticles, which enhance immune activity while minimizing off-target effects (87). Third, integrated nanoplatforms co-delivering immunotherapeutic and chemotherapeutic agents, such as nanoparticles combining PD-1 inhibitors with paclitaxel, achieve synergistic effects by inducing immunogenic cell death and bolstering immune responses (86). Fourth, multifunctional nanomaterials, including graphene, iron oxide, and peptide-modified nanoparticles, incorporate photothermal therapy, photodynamic therapy, and nanovaccines to amplify tumor antigen presentation and immune activation (88, 89). A notable example includes multifunctional nanoplatforms, such as small exosomes with high CD47 expression and cyclic arginine–glycine–aspartic modification, which effectively deliver short hairpin RNA targeting the m6A recognition protein YTH N6-methyladenosine RNA-binding protein 1 to achieve epigenetic and immune modulation in GC treatment (90). Moreover, exosome-engineered nanoparticles and DNA nanostructures enable personalized immunotherapy by tailoring treatments to the molecular characteristics of individual patients. Despite these advances, challenges such as long-term toxicity, safety concerns, and interpatient variability in therapeutic responses remain significant. Future research must address these limitations to accelerate clinical translation and unlock the full potential of nanotechnology-enhanced immunotherapy for GC (84).

#### 4.3.4 Clinical translation of immune checkpoint blockade and emerging therapeutic paradigms

The introduction of immune checkpoint inhibitors has revolutionized the treatment of advanced GC, with PD-1 inhibitors becoming key agents. Notably, nivolumab, a PD-1 inhibitor, has shown significant efficacy in patients with MSI-H tumors, substantially improving progression-free survival (PFS) and overall survival (OS) (91). Following this success, pembrolizumab combined with chemotherapy has yielded superior survival outcomes in PD-L1-positive patients while maintaining manageable immune-related toxicity (92). The effectiveness of these therapies, however, is highly dependent on biomarkers. For instance, atezolizumab continues to show efficacy in MSI-H and TMB-high patients, with a favorable safety profile (93), whereas durvalumab has demonstrated comparable benefits, particularly in MSI-H subgroups (94).

To address the limitations of monotherapy, combination therapies are gaining traction. The combination of ipilimumab and nivolumab has shown promising results in MSI-H patients, although some immune-related adverse effects have been observed, which are generally manageable (95). Additionally, sequential combinations of chemotherapy and atezolizumab in the neoadjuvant setting have achieved a 70% major pathological response rate, with 45% of patients experiencing pathological complete responses. These approaches were associated with manageable immune-related adverse events (10%) and no treatment-related delays, demonstrating the potential of combining immunotherapy with chemotherapy for enhanced tumor response in nonmetastatic GC (96). Further evidence highlights the potential of molecularly guided combinations, such as sitravatinib, a TAM kinase inhibitor that enhances PD-1 inhibitor efficacy through tumor microenvironment remodeling, achieving a 31.6% disease control rate (DCR) in gastroesophageal junction cancer (97).

Further advances in GC treatment are being driven by novel immunotherapies. HER-Vaxx, combined with chemotherapy, enhances OS and PFS in HER2-positive GC patients by inducing HER2-specific antibodies, promoting ADCC, and reducing FOXP3+ Tregs, offering a safe alternative to trastuzumab in cases of intolerable toxicity (98). Satricabtagene autoleucel, a CAR-T therapy targeting CLDN18.2, demonstrates high efficacy with a 91.8% DCR and 8.8-month median OS in advanced gastrointestinal cancers, offering a promising new option for refractory GC when combined with PD-1 inhibitors (99). While BVAC-B immunotherapy has shown limited clinical efficacy in HER2-positive GC patients, it activates immune cells and induces HER2-specific antibody responses, suggesting potential for enhanced efficacy in future combination therapies (100).The novel OBI-999, an antibody-drug conjugate targeting Globo H antigens, has shown good tolerability and disease stabilization in GC patients (101). Similarly, zolbetuximab, which utilizes antibody-dependent cell-mediated cytotoxicity, has demonstrated potent tumor growth inhibition in CLDN18.2-expressing GC and gastroesophageal junction cancers (102). Ivuxolimab and utomilumab, dual agonists targeting OX40 and 4-1BB, have shown acceptable safety and disease stabilization in GC patients, enhancing anti-tumor responses during dose escalation (103). Finally, CT041, a CLDN18.2-targeted CAR-T cell therapy, has demonstrated impressive efficacy with a 48.6% overall response rate and a 57.1% response rate in GC patients (99). This therapy is well-tolerated, with manageable immune-related adverse effects, and shows great promise for treating GC and other gastrointestinal cancers.

## 5 Conclusion

GC remains a major global health challenge due to its poor prognosis and limited therapeutic options, particularly in advanced stages. TME, and specifically TAMs, play a crucial role in GC progression, immune evasion, and treatment resistance. TAMs exhibit significant plasticity, shifting between pro-inflammatory M1 and immunosuppressive M2 phenotypes, with M2 TAMs predominating and correlating with worse clinical outcomes. Promising therapeutic strategies include inhibiting M2 polarization, reprogramming M2 macrophages into M1 phenotypes, and combining TAM-targeted therapies with ICIs. Advances in nanotechnology, metabolic reprogramming, and targeting key signaling pathways such as IL-6 and CCL2/CCR2 further enhance the potential of these approaches. However, challenges remain in addressing the spatial and functional heterogeneity of TAMs within the TME and achieving selective targeting without disrupting normal immune homeostasis (59). Continued research on TAM ontogeny, functional states, and interactions with the TME is critical for developing precise and effective therapies, with the potential to improve GC outcomes and inform treatment strategies for other cancers with complex microenvironments. Moving forward, future research should focus on identifying TAM subpopulation-specific molecular markers to guide precise therapeutic interventions, developing spatiotemporally controllable delivery systems to minimize off-target effects, and exploring multimodal combination therapies based on patient molecular subtypes (e.g., immune checkpoint blockade combined with metabolic regulators). These advances could not only improve GC treatment outcomes but also provide a model for targeting the microenvironment in other solid tumors.

## Glossary

CAFs: Cancer-associated fibroblasts
CCL2: C-C motif ligand 2
CCL2/CCR2: C-C motif ligand 2/C-C motif chemokine receptor 2
CCL5: C-C motif ligand 5
CSF-1/CSF-1R: Colony-stimulating factor 1/colony-stimulating factor 1 receptor
EGF: Epidermal growth factor
EMT: Epithelial-mesenchymal transition
GC: Gastric cancer
HER2: Human epidermal growth factor receptor 2
HIF1α: Hypoxia-inducible factor 1 alpha
ICIs: Immune checkpoint inhibitors
IFN-γ: Interferon gamma
IL-10: Interleukin-10
IL-12: Interleukin-12
IL-13: Interleukin-13
IL-24: Interleukin-24
IL-4: Interleukin-4
IL-6: Interleukin-6
IL-8: Interleukin-8
IM: Invasive margins
JAK/STAT: Janus kinase-signal transducer and activator of transcription signaling pathway
JAK/STAT: Janus kinase/signal transducers and activators of transcription signalling
MSI: Microsatellite instability
NF-κB: Nuclear factor kappa-B
NK: Natural killer
OS: Overall Survival
PD-1: Programmed death 1
PD-L1: Programmed Cell Death Ligand 1
PD-L1: Programmed cell death-ligand 1
PFS: Progression-Free Survival
PI3K/AKT/mTOR: Phosphoinositide 3-kinase/Protein Kinase B/Mechanistic Target of Rapamycin
SERPINE1: Cell-derived serpin family E member 1
SPI1+CD68+ macrophages: SPI1-positive CD68-positive Macrophages
STAT3: Activator of Transcription 3
STING: Stimulator of interferon genes
TAMs: Tumor-associated macrophages
TC: Tumor core
TCM: Traditional Chinese medicine
TGFβ1: Transforming growth factor-β1
TH1: T Helper 1 cells
TH2: T Helper 2 cells
TMB: Tumor mutational burden
TME: Tumor microenvironment
VEGF: Vascular endothelial growth factor
Wnt: Wingless-Related Integration Site
